# Cancer-Associated Fibroblasts from Hepatocellular Carcinoma Promote Malignant Cell Proliferation by HGF Secretion

**DOI:** 10.1371/journal.pone.0063243

**Published:** 2013-05-07

**Authors:** Chang-Chang Jia, Tian-Tian Wang, Wei Liu, Bin-Sheng Fu, XueFeng Hua, Guo-Ying Wang, Tuan-Jie Li, Xing Li, Xiang-Yuan Wu, Yan Tai, Jie Zhou, Gui-Hua Chen, Qi Zhang

**Affiliations:** 1 Department of Hepatic Surgery, The Third Affiliated Hospital of Sun Yat-sen University, Guangzhou, China; 2 Guangdong Provincial Key Laboratory of Liver Disease Research, Guangzhou, China; 3 Department of Medical Oncology, The Third Affiliated Hospital of Sun Yat-sen University, Guangzhou, China; 4 Cell-gene Therapy Translational Medicine Research Center, The Third Affiliated Hospital of Sun Yat-sen University, Guangzhou, China; 5 Department of Hepatobiliary Surgery, Nanfang Hospital, Southern Medical University, Guangzhou, China; University of Southern California, United States of America

## Abstract

Cancer-associated fibroblasts (CAFs) are reported to support tumorigenesis by stimulating angiogenesis, cancer cell proliferation, and invasion in most solid tumors. However, the roles of CAFs in the liver cancer microenvironment have not been thoroughly studied. In our previous study, we successfully isolated CAFs from hepatocellular carcinoma (HCC) (H-CAFs) and proved that H-CAFs suppressed the activation of NK cells and thereby created favorable conditions for HCC progression. In our present study, we found that the proliferation of MHCC97L and Hep3B cells was significantly promoted by treatment with conditioned medium from H-CAFs. Pathological analysis also revealed that H-CAFs increased the proportion of Ki-67 (+) malignant cells and prevented them from undergoing necrosis. Moreover, the concentration of hepatocyte growth factor (HGF) cytokine in the conditioned medium of H-CAFs was higher than conditioned medium from normal skin fibroblasts (NSFs). Anti-HGF significantly reduced the proliferation-promoting capability of H-CAFs. In addition, we found that the abundance of H-CAFs correlated positively with tumor size. These results indicate that H-CAFs are an important factor for promoting the growth of HCC *in vitro* and *in vivo*, and that HGF plays a key role in HCC proliferation induced by H-CAFs.

## Introduction

The marked influence of tumor stroma on the growth of malignant cancer cells has been demonstrated and investigated with enthusiasm in this era of targeted therapy [1], [2]. As a major component of the tumor stroma, increasing evidence has shown that cancer-associated fibroblasts (CAFs) are a significant modifier of cancer evolution[3], and they promote the tumorigenesis [4], progression [5], invasion [6] and chemoresistance [7] of cancer cells by various mechanisms. This is especially true for hepatocellular carcinoma (HCC) [8], [9] because most HCC cases may derive from liver cirrhosis [10]. CAFs are primarily resistant to chemotherapy and radiotherapy due to a small proportion of proliferating cells in contrast to malignant cells [11], which makes CAFs a potential source of tumor progression and relapse [12]. Targeted therapy toward CAFs has displayed promising anti-cancer efficacy [6], which has further reinforced the need for studying the relationship between CAFs and their hosts [13].

However, little is known concerning the CAFs in HCC (H-CAFs). H-CAFs were isolated and characterized in our previous study [8], which was comparable to the study of Antonio Mazzocca [9]. We found that H-CAFs educated NK cells to acquire a deactivated phenotype and create an unresponsive condition in tumors [8]. Additionally, the study by Antonio Mazzocca [9] indicated that lysophosphatidic acid (LPA) accelerates HCC progression by recruiting peri-tumor fibroblasts (PTFs) and promoting their transdifferentiation into myofibroblasts. However, that study did not determine the impact of CAFs on liver cancer cells in vitro. The role of H-CAFs in HCC is unknown. More studies are needed to obtain the full view of cross-talk between H-CAFs and HCC in the host.

CAFs are thought to be activated, which is characterized by the expression of α-smooth muscle actin (α-SMA), fibroblast activation protein (FAP), fibroblast surface protein (FSP), and vimentin [1]–[3]. This activation grants CAFs multiple functions in promoting cancer development, such as facilitating angiogenesis, epithelial–mesenchymal transition (EMT) [14], chemoresistance [5], [7], dysfunction of the local immune system [6], [8], and even more fundamentally, malignant cell proliferation [1]–[3]. However, the key molecular mechanism and functional signal pathway involved in these biological processes are poorly understood and far from fully investigated.

In the present study, we sought to elucidate the role of H-CAFs in promoting HCC cell proliferation and the underlying mechanism.

## Materials and Methods

### Patients and eligibility

We investigated a consecutive series of 43 patients with histologically confirmed hepatocellular carcinoma, who were admitted to the Third Affiliated Hospital of Sun Yat-sen University (Guangzhou, China) from November 2008 to August 2011. Patients were included with the following inclusion criteria: (a) pathologically confirmed as HCC, (b) age≥18, (c) treated with radical excision for HCC, (d) availability of CT data for tumor morphology, (e) lack of anticancer therapy, such as transcatheter arterial chemoembolization (TACE), sorafenib, chemotherapy and radiotherapy prior to surgery. The study was approved by the Clinical Research Ethics Committee of the Third Affiliated Hospital of Sun Yat-sen University, Guangzhou, China. A written informed consent was obtained from all the patients at the time of admission. The paraffin-embedded specimens and clinical data were retrieved for each patient.

### Isolation of primary fibroblasts from HCC specimens

Tumor samples were obtained from HCC patients, normal liver samples were obtained from hepatic hemangioma patients and foreskin samples were obtained from healthy donors from the Third Affiliated Hospital of Sun Yat-sen University. The diagnosis of HCC in all patients who underwent hepatolobectomy was confirmed by pathological and clinical tests. Samples were anonymously coded in accordance with local ethical guidelines (as stipulated by the Declaration of Helsinki), and written informed consent was obtained from patients and healthy volunteers. The work was conducted in strict accordance with the study design as approved by the Clinical Research Ethics Committee of the Third Affiliated Hospital at Sun Yat-sen University in Guangzhou, China.

H-CAFs were isolated from the cancerous region, and noncancerous fibroblasts (NFs) included three types of fibroblasts: peritumoral fibroblasts (PTFs) taken from tissue at least 2 cm distal to the outer margin of the cancer mass, fibroblasts derived from hepatic benign hemangioma as normal liver fibroblasts (NLFs) and fibroblasts obtained from foreskin as normal skin fibroblasts (NSFs). The tissues were minced and digested in Roswell Park Memorial Institute 1640 (RPMI 1640; Invitrogen, Carlsbad, CA) supplemented with 10% fetal bovine serum (FBS; Gibco-BRL, Grand Island, NY), 1 mg/ml collagenase type I (Sigma, St. Louis, MO) and 100 U/ml hyaluronidase (Sigma, St. Louis, MO) at 37°C for 6 to 8 hours, washed twice with PBS (Sigma, St. Louis, MO) and centrifuged at 450 g for 8 minutes each time. They were finally resuspended in RPMI 1640 supplemented with 10% FBS, 100 IU/ml penicillin, 100 µg/ml streptomycin, and then cultured at 37°C in a humidified 5% CO_2_ environment. Differential trypsinization was applied during sub-culturing to select for the growth of fibroblasts. The percentage of purified fibroblasts was 95% after 2–3 passages, which was determined by immunofluorescence, Western blotting and flow cytometry using antibodies against vimentin, cytokeratin, FAP, and α-SMA. Subsequent experiments were carried out using these cells within 3–10 passages.

### Cells and cell culture

The HCC cell line Hep3B (American Type Culture Collection, Manassas, VA, USA) was cultured in Minimum Essential Medium (MEM; Gibco-BRL, Grand Island, NY) supplemented with penicillin (100 U/ml), streptomycin (100 µg/ml) and 10% FBS. MHCC-97L cells (97L; Liver Cancer Institute, Zhongshan Hospital, Fudan University, Shanghai, China) were maintained in Dulbecco's Modified Eagle's Medium (DMEM; Gibco-BRL, Grand Island, NY) supplemented with penicillin (100 U/ml), streptomycin (100 µg/ml), L-glutamine (300 µg/ml) and 10% FBS. LO2 cells (obtained from our lab storage) were cultured in RPMI 1640 (Gibco-BRL, Grand Island, NY) supplemented with penicillin (100 U/ml), streptomycin (100 µg/ml) and 10% FBS. All cells were passaged at a ratio of 1∶4 every 4–5 days.

### Collection of conditioned medium and enzyme-linked immunosorbent assay (ELISA)

For the collection of conditioned medium from these fibroblasts, fibroblast cells were plated in 75 cm^2^ flasks, washed twice with PBS 4 days later, and incubated for 48 hours with serum-free DMEM. Then, the supernatant was harvested, centrifuged at 3,000 rpm for 5 mins, passed through a sterile Millipore 50 ml filtration system with a 0.45 µm polyvinylidenedifluoride membrane and stored in a −80°C refrigerator for later use.

To measure soluble cytokines in the conditioned medium, 2×10^5^ fibroblasts were plated in 6-well plates and cultured for 24 hours, and the conditioned medium was collected for ELISA. The same method was used to obtain conditioned medium from LO2 cells. The levels of human stromal cell–derived factor 1 (SDF-1), hepatocyte growth factor (HGF), transforming growth factor-beta (TGF-β) and epidermal growth factor (EGF) were determined by using human ELISA kits (R&D Systems, Minneapolis, MN) according to the manufacturer's instructions.

### Immunoblotting

Total protein was extracted using RIPA buffer after the wells were washed at least 3 times with PBS buffer. Proteins were separated by 12% SDS-PAGE, immunoblotted with an antibody against α-SMA (Abcam, Cambridge, MA), FAP (Abcam, Cambridge, MA), vimentin (Abcam, Cambridge, MA) and β-actin (Santa Cruz Biotechnologies, Santa Cruz, CA) and then visualized with the enhanced chemiluminescence system (GE, Buckinghamshire, UK).

### Immunofluorescence (IF)

The cells were fixed in ice-cold methanol (at 4°C for 15 mins), washed with PBS, blocked with 3% BSA, and incubated with primary antibodies for 1 hour at room temperature. Cells were immunostained for α-SMA, FAP, vimentin, FSP (Sigma, St. Louis, MO) and cytokeratin (Dako, Carpinteria, CA). The secondary antibodies were Alexa Fluor 488 goat anti-mouse IgG (H+L) and Alexa Fluor 546 goat anti-mouse IgG (H+L) (Molecular Probes, Grand Island, NY). Nuclei were stained with Hoechst 33342 (Sigma, St. Louis, MO). The images were viewed under a fluorescence microscope (LEICA DMI 4000B, Solms, Germany) and were analyzed by Leica application suite software (version 4.0).

### Immunohistochemistry

The tissues were fixed in 4% paraformaldehyde (PFA; Sigma, St. Louis, MO), embedded in paraffin and cut into 4 µm-thick sections. Endogenous peroxidase activity was blocked, antigen retrieval was performed, and the slides were stained with primary antibodies against Ki-67 (Abcam, Cambridge, MA), α-SMA and CD31 (Abcam, Cambridge, MA), which were used at the dilutions indicated by the manufacturer. Immunoreactions were detected by the Dako-Cytomation Envision HRP System (Dako, Glostrup, Denmark), and the sections were counterstained with hematoxylin (Sigma, St. Louis, MO). Negative controls were only stained with the secondary antibody.

H-CAFs were identified by α-SMA immunoreactivity. The population of H-CAFs was classified into high intensity or low intensity staining patterns according to previously described criteria [14].

### Cell proliferation assay

HCC cells (97L cells) were plated at a density of 2×10^3^ cells in triplicate in a 96-well plate (Corning Life Sciences, Lowell, MA). Conditioned medium containing 10% FBS from the fibroblast samples or from LO2 cells was added and exchanged for 4 to 5 days, and DMEM supplemented with 10% FBS was used as a control. Cell proliferation was measured by using the Cell Counting Kit-8 (Dojindo, Rockville, MD) according to the manufacturer's protocol.

### *In vivo* effect of H-CAFs on tumor growth

A liver cancer xenograft model was successfully established to evaluate the effect of H-CAFs on tumor growth in the present study. Four- to six-week-old male BALB/c nude mice were purchased from Wei Tong Li Hua Company (Beijing, China) and maintained in pressurized ventilated cages at the Vaccine Research Institute of Sun Yat-sen University. The 97L cells (5×10^6^) alone as a control or mixed with either CAFs (5×10^6^) or NFs (5×10^6^) were suspended in a 0.5 ml tube and injected subcutaneously (s.c) into nude mice. Tumor sizes were routinely measured with Vernier calipers every 3 days, and tumor volumes were calculated using the following formula: π/6×larger diameter×smaller diameter)^2^. The data were presented as a plot of mean tumor volumes versus time in days. All animal experiments were performed in accordance with the recommendations in the Guide for the Care and Use of Laboratory Animals of the Sun Yat-sen University and were approved by the Animal ethics committee of the Third Affiliated Hospital (Permit Number: 0021942).

### Statistical analysis

The data were presented as the mean ± SEM, and Student's t-test was used to compare the difference between two groups. *P* values less than 0.05 were regarded as statistically significant. Significant differences for continuous data in clinical characteristics between two groups (H-CAFs high intensity vs. H-CAFs low intensity) were compared using the Mann-Whitney test.

## Results

### Isolation, culture and characterization of H-CAFs

**Figure 1:**
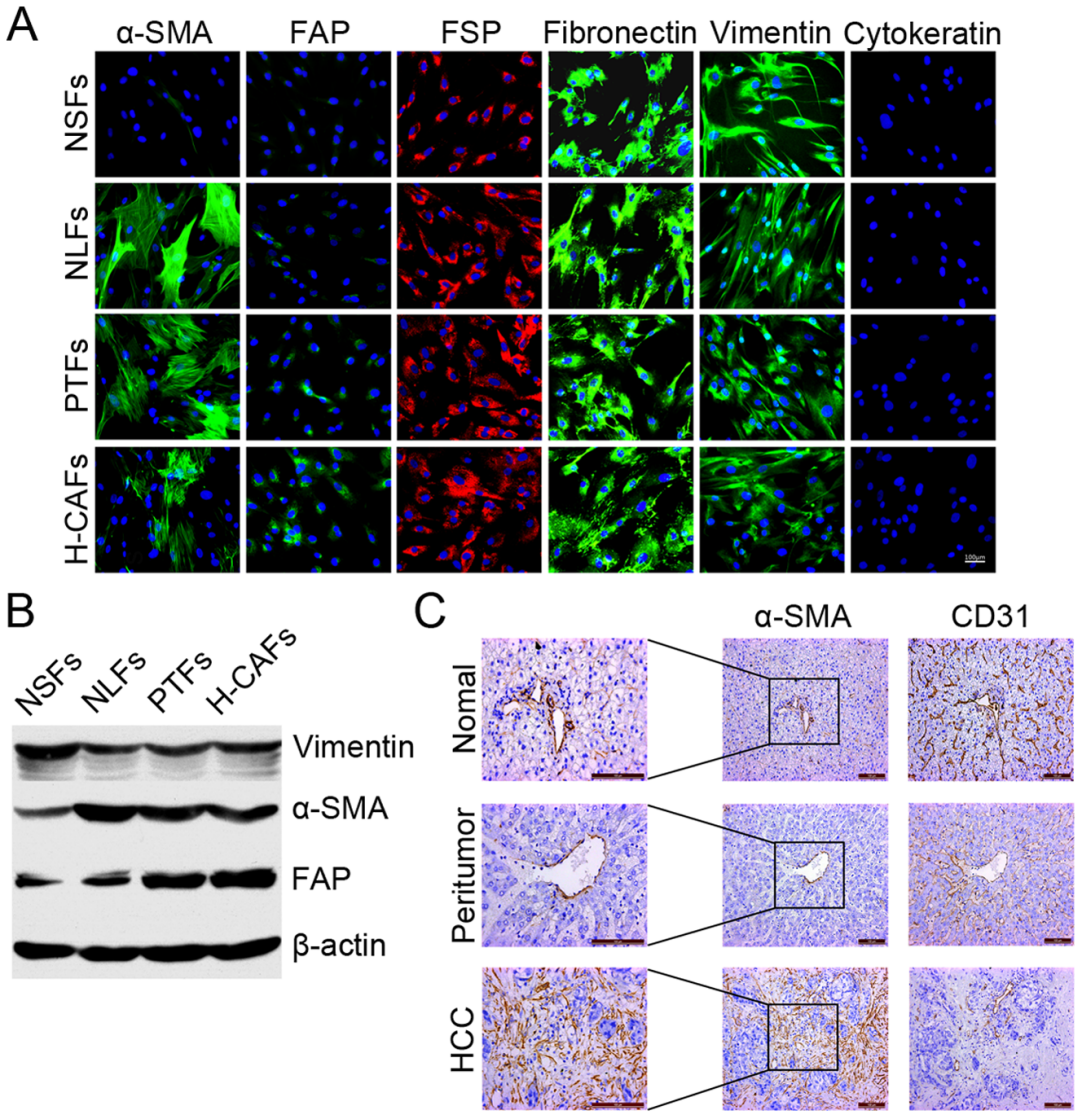
Characterization and distribution of H-CAFs *in vivo* and *in vitro*. (A) The expression of α-SMA, FAP, FSP, vimentin, fibronectin and cytokeratin in the four types of purified fibroblasts cultured for 3–10 passages was determined by immunofluorescent staining. All four fibroblast types showed high expression of the mesenchymal markers FSP, vimentin and fibronectin. NLFs, H-CAFs, PTFs and NLFs displayed high expression of α-SMA, which suggests that these fibroblasts exist in an activated state under cell culture conditions. However, another activation hallmark FAP was highly expressed in H-CAFs and PTFs but not in NSFs and NLFs. (B) Western blotting showed differences in α-SMA and FAP expression among the four fibroblasts. Consistent with the results of the immunofluorescent staining, H-CAFs, PTFs and NLFs displayed high expression of α-SMA. FAP was highly expressed in H-CAFs and PTFs but not in NSFs and NLFs. (C) The distribution of H-CAFs identified by α-SMA (+) CD31 (−) expression in each specimen from malignant, peri-tumor and normal liver regions was detected through immunohistochemistry in serial pathological sections. α-SMA expression was detected to confirm the presence of H-CAFs. In addition, CD31 expression was evaluated to exclude the presence of vascular endothelial cells, which co-express α-SMA and CD31. H-CAFs were more abundant in tumor tissue, compared with peri-tumor and normal liver tissue.

H-CAFs were isolated from primary tumor tissues and cultured according to the methods described in our previous study [8]. H-CAFs showed high-level expression of α-SMA, FAP, FSP, vimentin and fibronectin according to the immunofluorescence analysis (Fig. 1A), which was confirmed by Western blotting (Fig. 1B). Moreover, as the key feature of H-CAFs and activated fibroblasts, α-SMA expression was detected in primary tumor tissues using immunohistochemistry to confirm the presence of H-CAFs in tumor specimens. CD31 expression was evaluated to exclude the presence of vascular endothelial cells, which co-express α-SMA and CD31. The results demonstrated that H-CAFs were more abundant in tumor tissues compared with peri-tumor and normal liver tissue (Fig. 1C).

However, PTFs expressed a similar level of α-SMA, FAP, FSP, vimentin and fibronectin to H-CAFs *in vitro* (Fig. 1A), which was further confirmed by Western blotting (Fig. 1B). NLFs displayed a significantly lower expression of FAP compared with H-CAFs *in vitro*, and there was no significant difference in the other biomarkers (Fig. 1A and Fig. 1B). The expression of α-SMA and FAP was extremely low in NSFs in contrast with H-CAFs, which was demonstrated by immunofluorescence and Western blotting (Fig. 1A and Fig. 1B).

### H-CAFs promoted the proliferation of HCC cells both *in vivo* and *in vitro*

**Figure 2:**
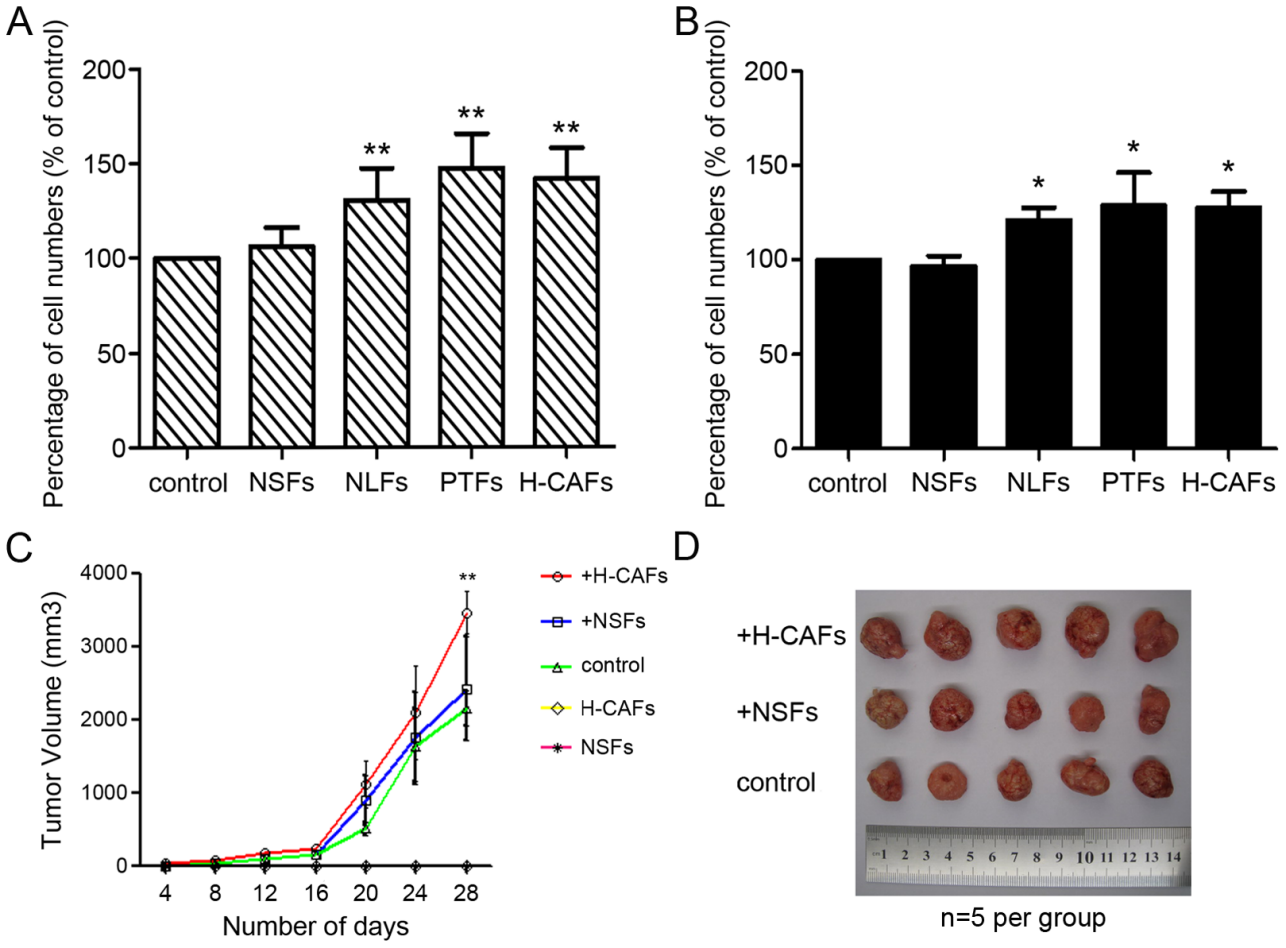
H-CAFs promoted the proliferation of HCC cells both in vivo and vitro. (A and B) 97L cells (A) and Hep3B cells (B) were cultured in conditioned medium from different fibroblasts, and the proliferation of malignant cells was assayed by CCK-8 analysis. NLFs, PTFs and H-CAFs significantly increased HCC cell proliferation relative to NSFs and the control group. (C and D) A BALB/c nude mouse xenograft model based on the co-injection of HCC cells with or without two fibroblast types (NSFs or H-CAFs) was used to investigate the *in vivo* interaction between H-CAFs and HCC cells. Tumor volumes of tumor nodes generated by the co-injection of HCC cells and H-CAFs were consistently significantly larger than those formed by HCC cells without co-injection of H-CAFs. NSFs did not significantly increase tumor growth relative to the control. In addition, fibroblasts did not generate tumors when injected alone. (C) Gross tumor specimens at the end of the experiment are shown. Larger HCC tumors are formed when HCC cells are co-injected with H-CAFs (n = 5 per group) (D). (* *P*<0.05; ** *P*<0.01).

CCK-8 tests demonstrated that the proliferation of 97L and Hep3B cells was significantly increased by treatment with conditioned medium from NLFs, PTFs and H-CAFs compared with conditioned medium from NSFs or the control group *in vitro*. These results suggest that H-CAFs possess a pro-proliferative effect on HCC cells (Fig. 2A and Fig. 2B).

To assess the contribution of H-CAFs and NSFs in terms of tumor growth *in vivo*, a liver cancer xenograft system was established as described in Methods section. Nude mice were subcutaneously injected with HCC cells, fibroblast cells, or with HCC cells and fibroblast cells. Tumor growth recorded for a maximum of 28 days after cell injection. Tumor size measurement showed that the implantation of pure H-CAFs or NSFs did not result in tumor formation. Furthermore, co-injection of H-CAFs contributed to the generation of larger tumors (HCC+H−CAFs *vs*. HCC, *P*<0.05, n = 5) in contrast to the co-injection of NSFs, which did not enhance tumor mass formation or growth (HCC+NSFs *vs*. HCC, *P*>0.05, n = 5) (Fig. 2C and Fig. 2D).

**Figure 3:**
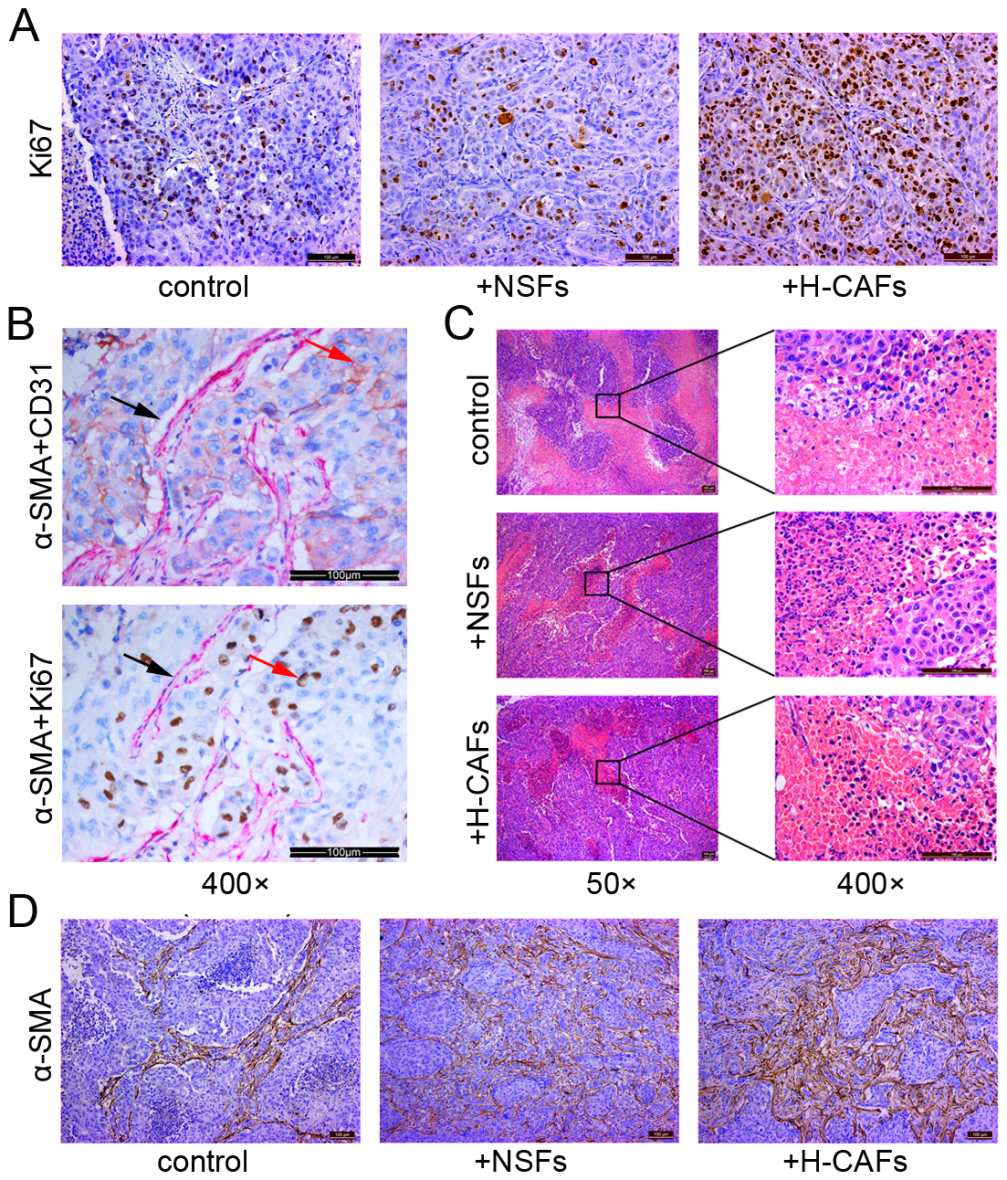
H-CAFs promoted malignant cell proliferation and protected malignant cells from necrosis in tumor xenograft, as revealed by immunohistochemical tests and H&E staining. (A) Ki-67 expression in HCC cells was substantially higher in tumor nodes formed by the co-injection of 97L cells and H-CAFs, which was in contrast with the results of tumors formed by the co-injection of 97L and NSFs or 97L cells alone. (B) Ki-67-positive cells were abundant near α-SMA (+) CD31 (−) fibroblasts within serial tumor sections. In samples that were double immunostained with α-SMA (red color, black row) and CD31 (brown color, red row), CD31 was not expressed in α-SMA-positive cells (Upper panel). When double immunostained with α-SMA (red color) and Ki-67 (brown color), a high incidence of tumor cells with Ki-67 expression (brown color, red row) was observed found in the vicinity of H-CAFs with α-SMA-positive staining (red color, black row) (Lower panel). (C) Necrosis was massively reduced in tumor nodes generated by the co-injection of fibroblasts (+NSFs or +H−CAFs), compared with tumors formed by 97L cell injection alone (control). (D) α-SMA expression was observed in all tumor xenografts, including the tumor nodes formed without fibroblast co-injection (control group).

Moreover, this promotion capability was confirmed by the percentage of Ki-67-positive tumor cells in tumor nodes. The Ki-67 (+) abundance directly reflects the growing fraction of tumor tissues. As expected, tumors formed by HCC and H-CAFs displayed strong and dense Ki-67 expression, while the other two groups showed moderate and scattered Ki-67 staining (Fig. 3A). Interestingly, a high incidence of Ki-67-positive cells was found around fibroblasts within serial sections, particularly in regions with massive necrosis, which further suggested the promotive role of fibroblasts on the survival of tumor cells (Fig. 3B).

This enhanced tumor node growth might be not only strengthened by proliferation promotion but also supported by avoiding necrosis. Necrosis occurred in tumor nodes of the three groups when the node diameter increased to a certain level. Interestingly, necrotic regions within the serial sections were much smaller in the tumor masses developed with fibroblasts (Fig. 3C). Furthermore, the remnant tumor cells in the necrotic regions were distributed around fibroblasts expressing α-SMA, which strongly indicated the survival role of fibroblasts for malignant cells (Fig. 3B).

Additionally, an interesting phenomenon was observed in our experiment. H-CAFs were also observed in tumor nodes formed by only HCC cells, which suggested that tumor cells may induce certain cells with unknown origin to transform into CAFs (Fig. 3D).

### HGF is involved in enhancing the proliferation of HCC cells

**Figure 4:**
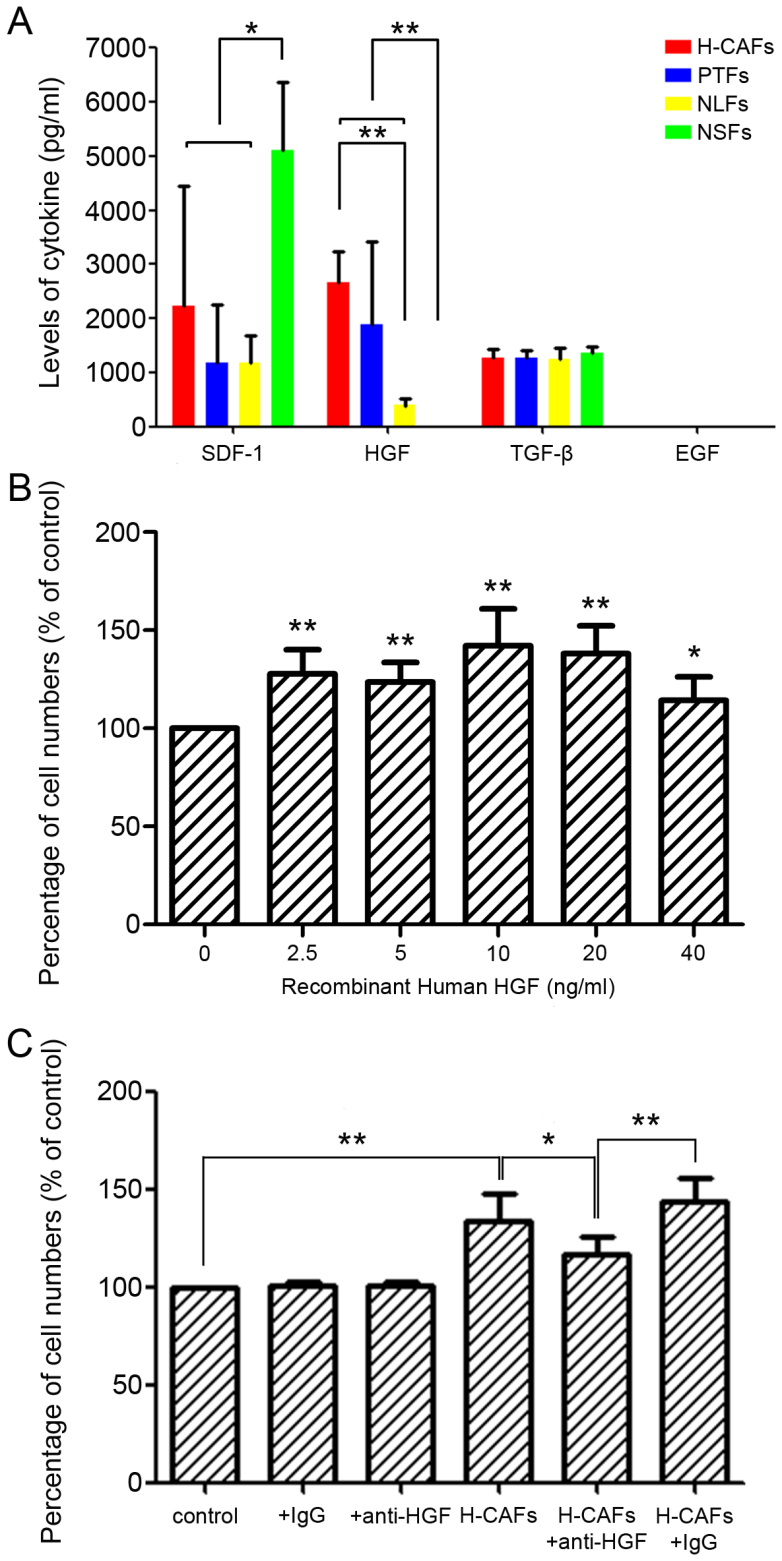
The proliferative enhancement of HCC cells by H-CAFs was partially mediated by HGF in a paracrine model. (A) The level of SDF-1, HGF, TGF-β and EGF in the conditioned medium derived from H-CAFs was determined by ELISA. H-CAFs and PTFs secreted HGF at significantly higher levels than NLFs, whereas NSFs secreted almost no detectable HGF. However, NSFs secreted a higher level of SDF1 than the other three fibroblast types. TGF-β and EGF secretion were similar for the four fibroblast types. (B) Cell proliferation increased upon exposure to different concentrations of HGF, as demonstrated by CCK-8 tests. HGF promoted HCC cell proliferation at all concentration levels. (C) The increase in proliferation of HCC cells as mediated by H-CAF conditioned medium was significantly antagonized by HGF-neutralizing antibody. Moreover, anti-HGF did not interfere with the proliferation of 97L cells. (* *P*<0.05; ** *P*<0.01).

It was highly indicated that H-CAFs might communicate with HCC cells through paracrine modes because H-CAF conditioned medium promoted the proliferation of HCC cells. Therefore, growth factors and chemokines may be the main mediators through which fibroblasts communicate with cancer cells. We measured the levels of four previously reported cytokines, SDF-1 [15], HGF [16], TGF-β [1], [3] and EGF, in the conditioned medium of H-CAFs. The ELISA showed that the HGF concentration in the conditioned medium of H-CAFs was the highest among the four types of fibroblasts. PTFs also demonstrated a high level of secretion of HGF, with no significant difference compared with H-CAFs. HGF secreted by NLFs was significantly decreased compared with H-CAFs and presented a decreasing tendency compared with PTFs. Moreover, NSFs did not secrete HGF. These results indicated that HGF secretion was correlated with the fibroblast type. However, this similar tendency was not observed in the concentrations of SDF-1, TGF-β or EGF secreted by the different fibroblasts (Fig. 4A).

It was interesting that the tendency of HGF production was consistent with that of tumor volume growth, which led to the hypothesis that HGF played a mediator role in HCC proliferation facilitated by H-CAFs (Fig. 2D and Fig. 4A). Accordingly, HGF was analyzed with CCK-8 tests to determine its proliferative potential. The results were positive for all of the groups at different concentrations of HGF (Fig. 4B). Furthermore, anti-HGF was administered to antagonize the effect of H-CAF conditioned medium on HCC cells and significantly reduced proliferation (Fig. 4C). Moreover, HGF was not detected in the conditioned medium of 97L cells, which excluded the HGF autocrine stimulative pathway of HCC cells. This finding was further evidenced by the fact that anti-HGF did not interfere with the proliferation of 97L cells (Fig. 4C). However, proliferation was not completely eliminated, which implied that HGF is not the only cytokine involved in the molecular mechanism of malignant cell proliferation mediated by H-CAFs.

### H-CAF-secreted HGF induced HCC cell proliferation

Furthermore, we analyzed the HGF level in conditioned medium of H-CAFs, normal hepatocytes (LO2) and HCC cells (97L and Hep3B) by ELISA to exclude the possibility of an HGF autocrine mechanism. Our results show a large difference in HGF secretion between H-CAFs and liver-derived cells regardless of the malignant nature. Moreover, HGF was not detected in LO2 conditioned medium (Fig. S1A). To examine the specific role of H-CAFs in HCC progression further, we compared the proliferation-promoting capability of H-CAFs to that of LO2 cells. Our results show that H-CAFs had a significantly stronger effect on HCC proliferation than LO2 cells. All of our findings suggest that HCC cell proliferation is not caused in an autocrine manner (Fig. S1B). Thus, our data suggest that HCC proliferation is promoted in a paracrine manner by HGF secreted by H-CAFs.

### Pathological activation is one of the key features of H-CAFs

**Figure 5:**
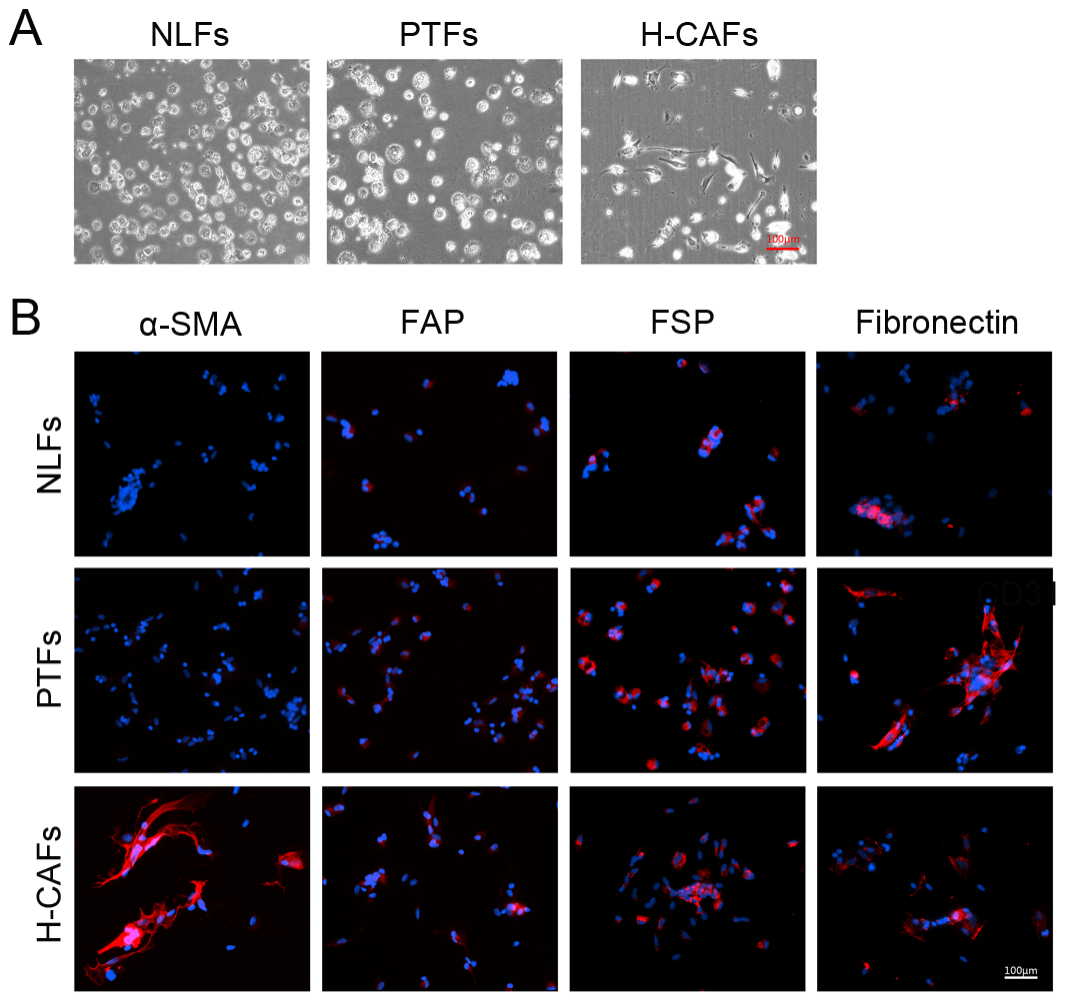
H-CAFs are pathologically activated in tumors, whereas NSFs and PTFs remain inactivated. (A) Microscope images of H-CAFs, PTFs and NSFs incubated for 24 hours. When originally isolated from the tumor specimens, PTFs and NLFs presented with a static, pericyte-like phenotype, whereas H-CAFs harbored an activated phenotype resembling myofibroblasts. (B) The expression of α-SMA, FAP, FSP and fibronectin in H-CAFs, PTFs and NSFs incubated for 24 hours was determined by immunofluorescence staining. α-SMA expression was remarkably higher in H-CAFs and relatively non-existent in the other fibroblast types when analyzed immediately following harvest from tumor specimens. This difference suggests the pathologically activated nature of H-CAFs and the relatively static nature of the other fibroblasts within the original tissues.

Similarities were found in the phenotype and proliferation enhancement among NLFs, PTFs and H-CAFs (Fig. 1A, Fig. 1B, Fig. 2A and Fig 2B) in *in vitro* experiments, including α-SMA expression. However, fibroblasts derived from regions of tumor tissue, peri-tumor tissue and normal liver tissues expressed different levels of α-SMA, the specific marker for fibroblast activation. This inconsistency in results might be caused by the fact that fibroblasts would transform from a static, pericyte-like phenotype to an activated phenotype resembling myofibroblasts after a few days of culture *in vitro*. Compared to a previous study, normal fibroblasts were activated after 1 week of culture *in vitro*, which was characterized by α-SMA expression (Fig.1A and Fig. 1B). Thus, it was logical to hypothesize that PTFs and NLFs were static in tumors and activated during culture. Furthermore, H-CAFs were relatively activated in tumors. Consistent with this hypothesis, the present study showed that PTFs and NLFs were “pericyte like”, while H-CAFs were “myofibroblast like” when they were originally isolated from tumor specimens (Fig. 5A). To further test this hypothesis, immunofluorescence was used to detect α-SMA expression among different types of fibroblasts incubated for 24 hours after the isolation of original tissue. As shown in figure 5B, H-CAFs were in an activated phase, as indicated by α-SMA expression, while PTFs and NLFs presented a resting phenotype, as demonstrated by negative α-SMA staining, indicating that pathological activation may be the main reason for the difference between normal fibroblasts and H-CAFs.

### The presence of H-CAFs positively correlates with tumor size

**Figure 6:**
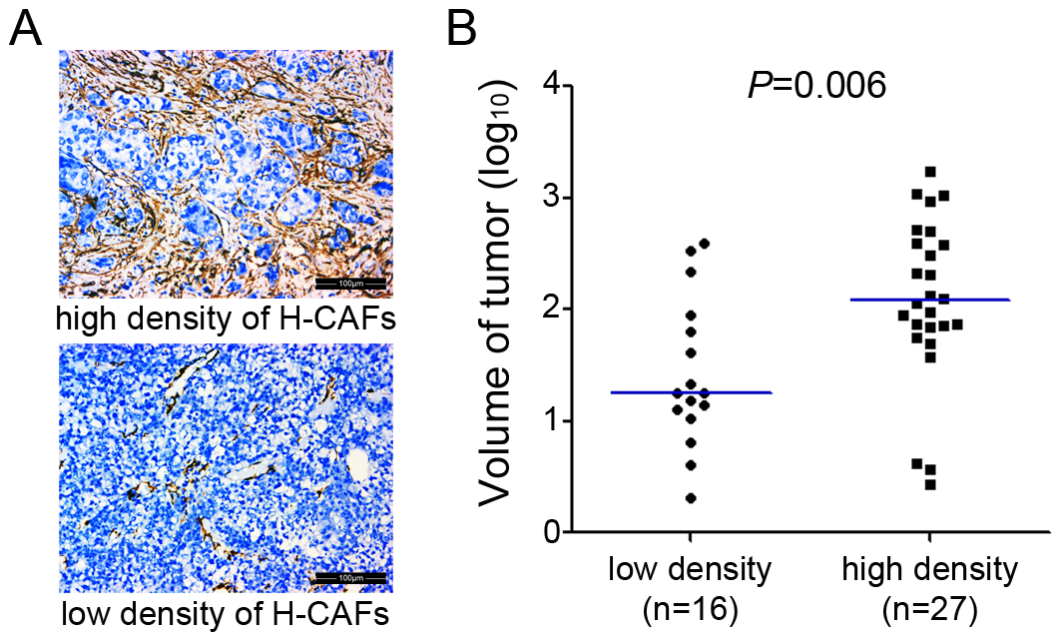
The abundance of H-CAFs positively correlated with tumor volume in human HCC samples. (A) The abundance of H-CAFs was dichotomized into two levels, high density and low density. (B) Tumor volumes of the high-density group (n = 27) were significantly higher than tumor volumes of the low-density group (n = 16, *P* = 0.006).

To determine the clinical importance of H-CAFs in the context of tumor growth, we investigated the presence of H-CAFs within tumor samples obtained from 43 patients by α-SMA immunoreactivity. Our results show that twenty-seven patient samples displayed a high level of staining for H-CAFs, whereas the remaining sixteen samples presented with a low level of staining for H-CAFs (Fig. 6A). Notably, the aggregation of H-CAFs was significantly associated with larger tumor size (Fig. 6B and Table S1), which suggests a positive correlation between the relative abundance of H-CAFs and tumor growth on the clinical level.

## Discussion

Hepatocellular carcinoma (HCC) is the fifth most prevalent form of cancer and the third leading cause of cancer–related deaths, accounting for 90% of all cases of primary liver cancer. The role of the tumor microenvironment during carcinogenesis has been largely studied as a dynamic system orchestrated by inflammatory cells, including cancer cells. The microenvironment of HCC is composed of fibroblasts, invading inflammatory cells, endothelial cells (ECs), pericytes adjacent to the ECs, and extensive extracellular matrix (ECM) components. Carcinoma-associated fibroblasts (CAFs) are one of the most crucial components of the HCC microenvironment. In our previous study, we successfully isolated specific carcinoma-associated fibroblasts of HCC (H-CAFs) from primary HCC tissues and proved that H-CAFs suppressed the activation of NK cells and created a favorable immunosuppressive shield for HCC cells. In our present study, we studied whether H-CAFs could enhance the growth of liver cancer cells.

Fibroblasts are often identified by their spindle-shaped morphology, their ability to adhere to plastic, and their lack of markers indicating other cell types [1]. However, universally specific and applicable molecular markers for fibroblasts are poorly defined. FSP, vimentin and fibronectin are considered acceptable molecular markers for identifying fibroblasts and are generally employed in studies on CAFs [1]. In the present study, all of the fibroblast lineages expressed FSP, vimentin and fibronection, which indicated their common identity as fibroblasts. Meanwhile, normal fibroblasts can acquire an activated phenotype induced by various pathologic conditions, such as tissue injury, chronic inflammation, and malignant diseases [17]. Activated fibroblasts are characterized by α-SMA and FAP expression and increased multiple biologic functions, such as the increased secretion of certain cytokines and matrix metalloproteinase (MMP) [1]. Activated fibroblasts in tumor tissues are considered CAFs [1], [3], and our results are consistent with the identification of activated H-CAFs as these cells expressed α-SMA and FAP, whereas NSFs expressed neither of these two markers. Furthermore, the molecular hallmarks of H-CAFs in the present study and in our previous report were consistent with a study performed by Antonio Mazzoca [9], who also identified H-CAFs simultaneously with but independent of our group. Our data has shown that H-CAFs remain stable regarding morphology and molecular characteristics through at least ten generations, which facilitates functional research regarding the effects of H-CAFs on malignant cells.

Our present study proved that H-CAFs possessed the capability to promote HCC cell proliferation *in vivo* and *in vitro*. Not only did the CCK-8 assay show the malignant cell proliferation promotion capability of H-CAFs, the xenograft model of nude mice also demonstrated tumor node growth enhancement by H-CAFs. Most importantly, the ability of H-CAFs to support the growth of tumor cells was fundamentally supported and further confirmed by our clinical data. The relative abundance of H-CAFs positively correlated with tumor size in the human HCC samples, which suggests that H-CAFs enhance tumor formation in human HCCs in a clinically important manner.

However, the increase in cell proliferation induced by NLFs, PTFs and H-CAFs was equal according to the CCK-8 assays in this study. The increased induced proliferation of NLFs was indicated by the fact that activated hepatic stellate cells could increase HCC cell proliferation [16]. Because all types of fibroblasts employed in the proliferation tests were considered to be activated, with α-SMA and FAP expression [1], [17], we suspect that the activation of the fibroblasts but not necessarily the origin of the fibroblasts accounted for the increase in HCC proliferation.

CAFs stimulate malignant cell proliferation by providing different types of growth factors and cytokines in a context-dependent manner [18], such as HGF [19], members of the epidermal growth factor family [20], fibroblast growth factor (FGF) [21], [22], Wnt families [23], SDF-1 (CXCL12) [15], fork-head box F1 (FoxF1) [24] and IL-6 [25]. The results from our present study show that HGF predominantly mediates the proliferative enhancement of HCC cells by H-CAFs. Our results show that HGF acts in a pro-tumorigenic manner during HCC carcinogenesis and promotes the proliferation of HCC cell lines, which is consistent with results from previous studies [16], [26]. The administration of anti-HGF partially reversed the cell growth promoting effect of H-CAF conditioned medium on HCC cells. However, HGF did not appear to be the exclusive mediator of this proliferative enhancement because some increase in growth was observed even in the presence of anti-HGF. Interestingly, HGF treatment displayed an inconsistent influence on cell proliferation. HGF stimulates the growth of some tumor cell lines, whereas HGF inhibits the growth of a number of other tumor cell lines [27]. Importantly, our results did not show a dose-dependent manner of proliferation in some cell lines, which is similar to previous studies [28]. The underlying causes of this paradox could involve differences in the expression of receptors that are responsive to HGF in cells of various origins. HGF stimulates the activation of PI3K, pp60^c-src^, phospholipase C_γ_, c-Met and Ras-ERK signaling pathways. Cells harboring different dominant signaling pathways could react differently to HGF. The 97L cell line employed in our study has been reported to express the c-Met receptor [29], which suggests that the HGF/c-Met pathway is most likely the main mechanism through which H-CAFs induce HCC cell proliferation at least in 97L cells. In summary, HGF appears to play a substantial role in the complex mechanisms involved in the proliferative induction of HCC by H-CAFs.

In addition to their ability of secreting growth factors to enhance HCC cell growth, H-CAFs also support cancer cell survival in severe environments. According to our results from the xenograft model, a fraction of malignant cells avoided death in a region with massive necrosis, possibly with the help of H-CAFs expressing α-SMA (+) CD31 (−). This specific phenomenon strongly indicated that the fibroblasts served as a scaffold and possessed cytoskeletal elements that facilitated decreases in damage. H-CAFs either modified the local environment with low pH and hypoxia caused by insufficient angiogenesis or mediated HCC cell metabolic pathways. Moreover, CAFs preferred to undergo anaerobic glycolysis even in the presence of oxygen during the proliferation of cancer cells. Furthermore, CAFs are able to turn on complementary metabolic pathways to buffer and recycle products of anaerobic metabolism, facilitating the maintenance of cancer cell survival [30]. Thus, H-CAFs might enhance malignant cell survival by modifying local severe conditions.

Interestingly, in contrast with previous studies revealing that elevated SDF-1/CXCL12 secretion by stromal fibroblasts promoted the tumor growth of human breast carcinomas [15], SDF-1 secretion was significantly lower in H-CAFs than NSFs, which indicated the difference between CAFs and different malignancies. Meanwhile, the TGF-β generation of H-CAFs was equal to other types of fibroblasts. These results do not refute the biologic function of the two cytokines in mediating H-CAF behaviors in HCC but indicate their relatively subordinate role in the cross-talk between HCC cells and H-CAFs. Because HCC is unique among all malignancies because of liver cirrhosis, the underlying molecular relationship between cancer cells and CAFs might differ from other malignancies.

The origin of H-CAFs is still unclear [3], [13], [31], and mesenchymal stem cells [21] and EMT[32] might be the sources. These views are further supported by the histological findings in the mouse model experiments in the present study. α-SMA (+) fibroblasts were also observed in tumor nodes formed by HCC cells without co-injected fibroblasts, which suggested that certain cells from unknown origin might transform into H-CAFs in the presence of HCC cells. Furthermore, our results partially indicated that H-CAFs were pathologically activated and transformed from certain cells by malignant cells or an inflammatory microenvironment.

In summary, the present study further supports the impact of H-CAFs on HCC proliferation *in vitro* and *in vivo*, with HGF being implicated as an important mediator. This interaction may be an interesting tumor cell differentiation-independent target for therapy. Furthermore, the quantification of H-CAFs in HCC might serve as a prognostic marker.

## Supporting Information

Figure S1 **ELISA analysis shows that HGF is secreted by H-CAFs but not normal hepatocytes or HCC cells.** Additionally, the proliferation of HCC cells in the presence of H-CAFs was increased to a greater extent than when cultured in the presence of normal hepatocytes. (A) The HGF level in the conditioned medium of these cells including H-CAFs, normal hepatocytes (LO2) and HCC cells (97L and Hep3B) was analyzed by ELISA. The concentration of HGF was dramatically higher in H-CAF conditioned medium. Furthermore, HGF was not detectable or remained at a low level in the conditioned medium derived from LO2, 97L and Hep3B cells. (B) The proliferation of HCC cells (97L cells and Hep3B cells) in the H-CAF group was compared to the LO2 group. Our results show a significantly stronger proliferation-promoting capability of the H-CAFs, compared with the LO2 cells. (* *P*<0.05; ** *P*<0.01).
(TIF)

Table S1 **Abundance of H-CAFs in relation to tumor volume.**
(DOCX)
