# Supplementary figures and images for: Cancer-Associated Fibroblasts from Hepatocellular Carcinoma Promote Malignant Cell Proliferation by HGF Secretion
Source: PLoS One. 2013 May 7;8(5):e63243. doi: 10.1371/journal.pone.0063243 (PMC3647063; doi:10.1371/journal.pone.0063243)

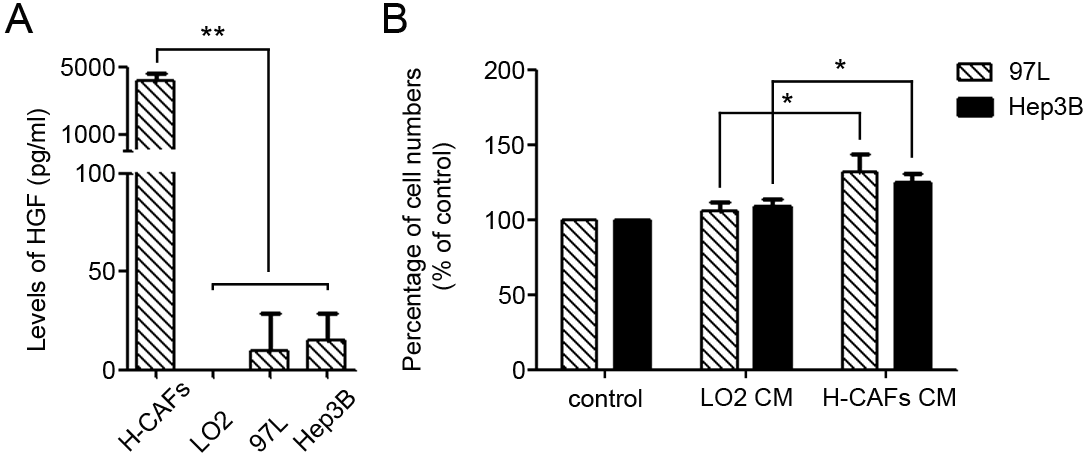

Supplement: Figure S1 — ELISA analysis shows that HGF is secreted by H-CAFs but not normal hepatocytes or HCC cells. Additionally, the proliferation of HCC cells in the presence of H-CAFs was increased to a greater extent than when cultured in the presence of normal hepatocytes. (A) The HGF level in the conditioned medium of these cells including H-CAFs, normal hepatocytes (LO2) and HCC cells (97L and Hep3B) was analyzed by ELISA. The concentration of HGF was dramatically higher in H-CAF conditioned medium. Furthermore, HGF was not detectable or remained at a low level in the conditioned medium derived from LO2, 97L and Hep3B cells. (B) The proliferation of HCC cells (97L cells and Hep3B cells) in the H-CAF group was compared to the LO2 group. Our results show a significantly stronger proliferation-promoting capability of the H-CAFs, compared with the LO2 cells. (* P<0.05; ** P<0.01). (TIF) [file pone.0063243.s001.tif]
