# Supplementary material for: Cancer-Associated Fibroblasts from Hepatocellular Carcinoma Promote Malignant Cell Proliferation by HGF Secretion
Source: PLoS One. 2013 May 7;8(5):e63243. doi: 10.1371/journal.pone.0063243 (PMC3647063; doi:10.1371/journal.pone.0063243)
Supplement: Table S1 — Abundance of H-CAFs in relation to tumor volume. (DOCX) [file pone.0063243.s002.docx]

| Table S1. Abundance of H-CAFs in relation to tumor volume | | |
| --- | --- | --- |
| H-CAF abundance | Tumor volume (median, range, cm^3^) | *P-*value |
| High density | 120.12 (2.69-1713.15) | 0.006 |
| Low density | 17.78 (2.03-393.76) |  |
